# Energy balance in cyclists on plant‐based diets during a 30‐day, 4300‐km ride across Canada: Two case studies

**DOI:** 10.14814/phy2.70629

**Published:** 2025-11-02

**Authors:** Sarah A. Purcell, Edward L. Melanson, Seth A. Creasy, Matthew Barros, Stephanie Ramage, Sarah A. Craven, Carla M. Prado

**Affiliations:** ^1^ Division of Endocrinology, Department of Medicine, Faculty of Medicine University of British Columbia Vancouver British Columbia Canada; ^2^ Centre for Chronic Disease Prevention and Management, Southern Medical Program, Faculty of Medicine University of British Columbia Kelowna British Columbia Canada; ^3^ School of Health and Exercise Sciences, Faculty of Health and Social Development University of British Columbia Okanagan Kelowna British Columbia Canada; ^4^ Division of Endocrinology Metabolism and Diabetes, School of Medicine University of Colorado Anschutz Medical Campus Aurora Colorado USA; ^5^ Division of Geriatric Medicine, School of Medicine University of Colorado Anschutz Medical Campus Aurora Colorado USA; ^6^ Anschutz Health and Wellness Center, School of Medicine University of Colorado Anschutz Medical Campus Aurora Colorado USA; ^7^ Human Nutrition Research Unit, Department of Agricultural, Food, and Nutritional Science, Faculty of Agricultural, Life, and Environmental Sciences University of Alberta Edmonton Alberta Canada

**Keywords:** aerobic exercise, bicycling, endurance exercise, energy metabolism, nutrition intake

## Abstract

The popularity of ultra‐endurance events and plant‐based diets highlights the importance of understanding the energetics of athletes with diverse dietary preferences. This study examined energy balance in two recreational cyclists on plant‐based diets (male, 41 years; female, 38 years) during a 30‐day cross‐Canada ride. Resting energy expenditure was measured via whole‐room indirect calorimetry before and after the ride. Total energy expenditure (TEE) was assessed using doubly labeled water during the first and last weeks of the ride and used to calculate physical activity energy expenditure (PAEE) and physical activity level (PAL). Body composition was assessed with deuterium dilution, and dietary intake was recorded using food scales, written records, and photographs. Cyclists averaged 154.8 ± 24.0 km/day early and 118.2 ± 25.3 km/day late in the ride. Body weight showed minimal variation, but the male's fat mass decreased by 2.3 kg and fat‐free mass increased by 1.4 kg. Both cyclists increased energy intake (female: +421 kcal/day; male: +761 kcal/day), with protein intake >2.3 g/kg/day. TEE increased in the female (+683 kcal/day) but remained relatively stable in the male (−137 kcal/day), driven by PAEE. PAL remained high (female: 3.71–4.11; male: 3.76–3.94). These findings highlight the high energy demands of ultra‐endurance cycling and the need for tailored strategies, particularly for athletes on plant‐based diets.

## 1 INTRODUCTION

Participation in ultra‐endurance events – defined as either exercising for more than 6 h or running more than 42.2 km – has increased in recent years (Scheer, 2019; Zaryski & Smith, 2005). These events test the limits of human performance and offer insight into physiology and metabolism. Nutrition plays a key role in supporting energy balance and optimizing performance before, during, and after such efforts (Thomas et al., 2016). This is especially important for the growing number of vegetarian and vegan athletes, whose plant‐based diets may be higher in fiber and lower in energy and protein, which may pose challenges for meeting nutritional needs during extreme endurance efforts (Ghaffari et al., 2022; West et al., 2023).

Ultra‐endurance cycling tests both physical and metabolic limits through prolonged, intense exertion over extended durations. While previous studies have examined nutritional and physiological responses to such events, most have focused on male athletes and single‐day efforts (Chlíbková et al., 2014; Geesmann et al., 2014; Guex et al., 2020; Moyen et al., 2015; Stuempfle et al., 2003; Wirnitzer & Faulhaber, 2007). However, none have specifically assessed outcomes in athletes on plant‐based diets. In light of these knowledge gaps, this study assessed dietary energy and macronutrient intake, total energy expenditure (TEE), and body composition of two cyclists on plant‐based diets during a 30‐day ride across Canada.

## 2 METHODS

### 2.1 Study design and subjects

Two recreational cyclists (one female, one male) participated in a cross‐Canada ride from Halifax to Vancouver, spanning July 1 to July 30, 2024. Both cyclists consumed a plant‐based diet excluding all animal products with the exception of honey. The cyclists were accompanied by a single support person but carried their own food and beverages between self‐directed stops. The female cyclist had been using a combined oral contraceptive containing 10 mcg of ethinyl estradiol and 1 mg of norethindrone acetate until 1 week prior to the ride, at which point she discontinued the oral contraceptive and had a hormonal intrauterine device inserted. Based on self‐reported menstrual cycle history, the female cyclist was likely in the late follicular phase during pre‐ride assessments, the late follicular to early luteal phase during the early phase of the ride, the early to mid‐follicular phase during the late phase of the ride, and the mid‐follicular phase during post‐ride assessments.

Baseline assessments included resting energy expenditure (REE) using a whole‐room indirect calorimeter and total body water (TBW) estimated using bioelectrical impedance analysis (BIA) at the Human Nutrition Research Unit at the University of Alberta 4 days before and 2 days after the ride. Doubly labeled water (DLW) was used to estimate TEE and fat‐free mass (FFM) during two measurement periods: Days 1–7 (“early ride”) and 23–30 (“late ride”). Fat mass (FM) was calculated as body weight–FFM during each period. Dietary intake was assessed on Days 1–4 and 23–26 using weighted food records. All details regarding these assessments are described in Data S1. Data on daily distance, elevation, and duration of cycling was recorded using a Garmin Edge 1040s, configured to automatically pause data recording during periods of inactivity. The University of Alberta Research Ethics Board approved the study, and both participants provided informed written consent.

## 3 RESULTS

### 3.1 Performance, anthropometric, and body composition data

At baseline, the female participant was 38 years of age (body mass index: 22.5 kg/m^2^) and the male participant was 42 years of age (body mass index: 26.2 kg/^2^). Throughout the ride, the cyclists each rode a total of 4316 km (143.9 ± 29.5 km/day) and 213.76 hours (7.25 ± 1.36 h/day), with a total combined net elevation of 696 m. Mean daily distance (−37 km/day), duration (−1 h, 8 min [i.e., −01:13] h/day), and net elevation decreased (−155 m/day) between the early and late phases of the ride, (Table 1).

**TABLE 1:** Daily cycling duration, distance covered, and net elevation gain in early and late ride.

Day of ride	Date	Cycling time, hours	Distance, km	Net elevation gain, m
Early ride				
1	July 01	6.82	163.8	3
2	July 02	8.90	199.4	30
3	July 03	8.29	155.3	−6
4	July 04	7.70	150.3	72
5	July 05	7.75	150.8	25
6	July 06	7.24	114.5	540
7	July 07	7.87	164.7	−287
8	July 08	6.68	140.1	21
Mean ± SD		7.66 ± 0.74	154.8 ± 24.0	50 ± 227
Late ride				
23	July 23	4.87	100.9	150
24	July 24	6.75	110.8	−65
25	July 25	6.11	82.0	289
26	July 26	7.18	136.1	−356
27	July 27	8.51	167.3	−578
28	July 28	7.26	123.2	514
29	July 29	6.20	111.9	313
30	July 30	5.35	113.5	−1107
Mean ± SD		6.53 ± 1.16	118.2 ± 25.3	−105 ± 544

TBW obtained from the BIA (measured 2–4 days before and after the ride) and DLW showed good agreement at both time points in both athletes. In the female cyclist, TBW was 39.0 kg via BIA and 38.0 kg via DLW at the early ride measurement, and 38.5 kg via BIA and 38.2 kg via DLW at the post‐ride measurement. In the male cyclist, TBW was 60.4 kg via BIA and 59.6 kg via DLW at the early ride measurement, and 59.7 kg via BIA and 60.6 kg via DLW at the post‐ride measurement.

Both athletes experienced a body weight loss of <2%. The female cyclist maintained relatively stable body weight (63.3 kg on Day 1 vs. 62.9 kg on Day 23), while the male cyclist exhibited a slight reduction (95.1 kg on Day 1 vs. 93.3 kg on Day 23). For the female athlete, both fat mass (11.3 kg at both timepoints) and fat‐free mass (52.0–52.4 kg, ∆ 0.4 kg) showed negligible change. In contrast, the male athlete experienced a decrease in fat mass (13.5–11.6 kg, ∆‐1.9kg) and a slight increase in fat‐free mass (81.6–83.0 kg, ∆ 1.4 kg).

### 3.2 Dietary intake

**FIGURE 1:**
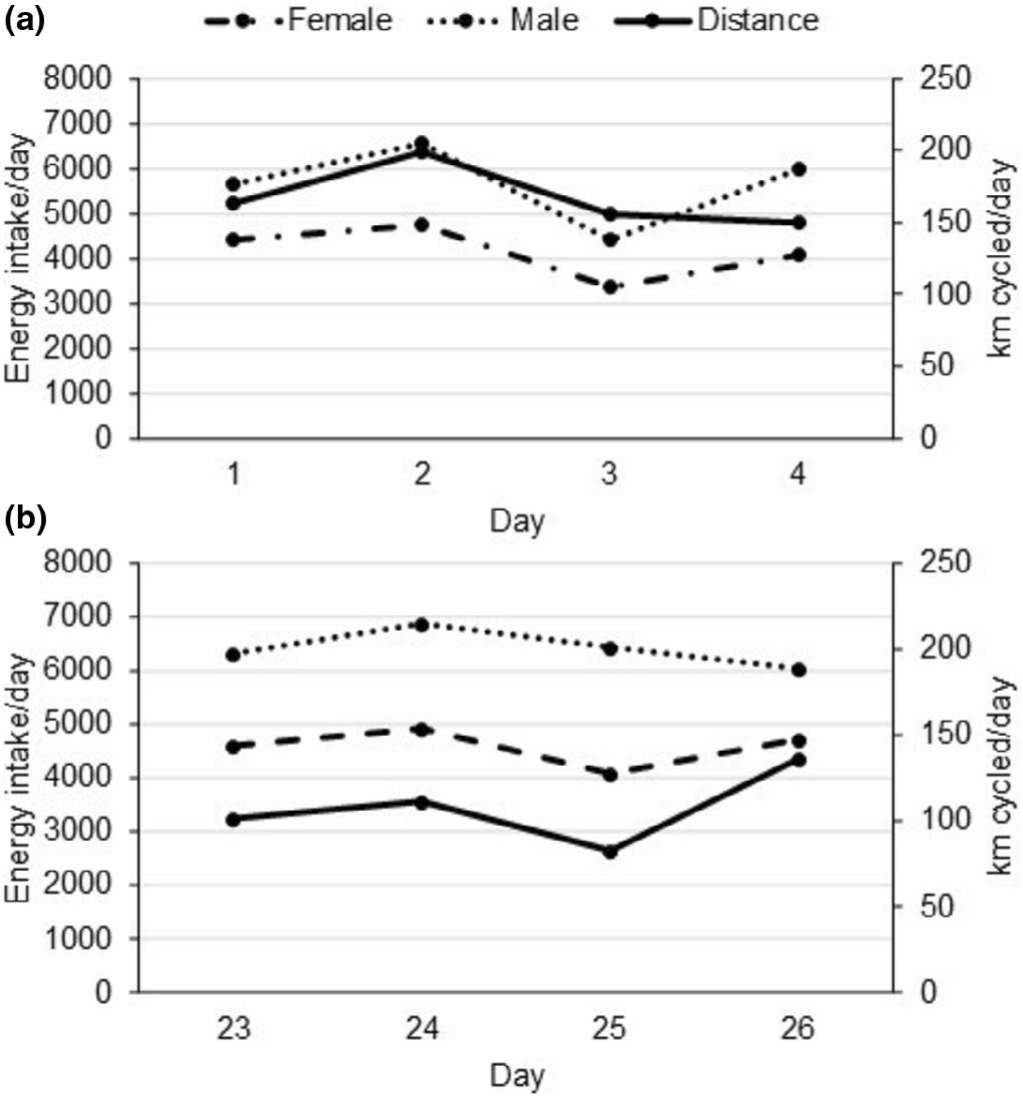
Daily energy intake and distance cycled in the early (a) and late (b) phases of the ride.

According to food records, both cyclists increased mean daily energy intake between early and late phases of the ride (∆: female: 421 kcal/day; male: 761 kcal/day), (Table 2). Both athletes consumed >2.3 g/kg body weight of protein per day. The proportion of energy from carbohydrates increased while the proportion of energy from protein and fat decreased across time. An example of food consumed during each phase of the ride is provided in Data S2. Daily dietary energy intake generally followed the same trend in daily distance covered in both phases of the ride, (Figure 1).

**TABLE 2:** Early and late ride self‐reported dietary intake.

	Athlete 1‐female		Athlete 2‐male	
	Early ride	Late ride	Early ride	Late ride
Energy (kcal/day)	4154	4575	5651	6412
Energy (kcal/kg/day)	65.6	72.7	59.4	68.7
Protein (g/day)	158	151	244	212
Protein (g/kg/day)	2.5	2.4	2.6	2.3
Protein (% energy intake)	15.1	13.2	17.3	13.2
Carbohydrate (g/day)	591	689	754	961
Carbohydrate (% energy intake)	57.0	60.3	53.5	59.8
Fat (g/day)	156	152	211	227
Fat (% energy intake)	33.9	30.0	33.5	31.9

### 3.3 Energy expenditure

**FIGURE 2:**
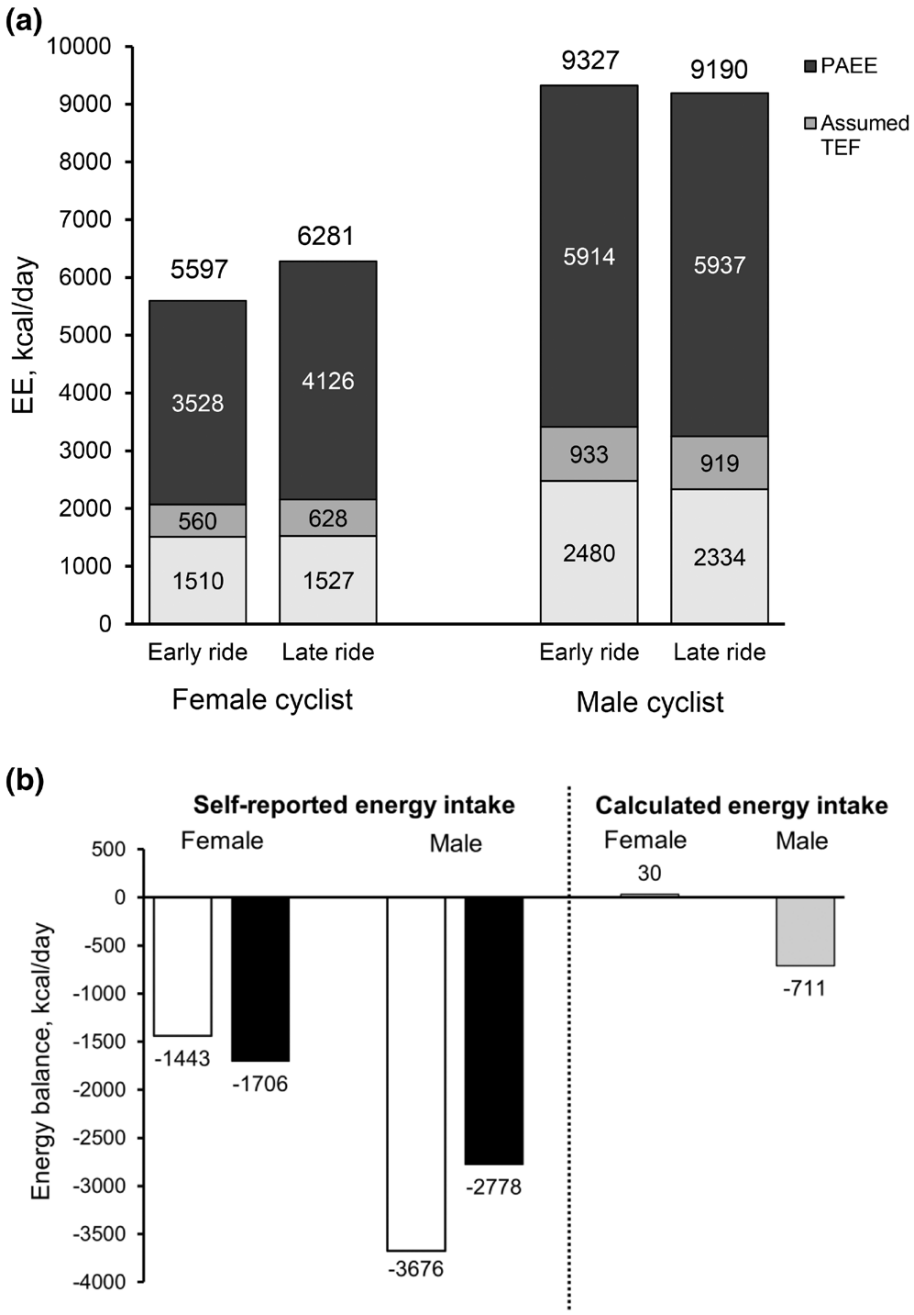
Total energy expenditure and its components (a) and energy balance with self‐reported and calculated energy intake (b) in the early and late phases of the ride. (a) The numbers above each column are total energy expenditure (TEE), calculated using the intercept approach. TEE using the plateau approach is as follows: Female early ride: 5681 kcal/day, late ride: 6239 kcal/day; male early ride: 9530 kcal/day, late ride: 9190 kcal/day. TEE: Total energy expenditure; PAEE: Physical activity energy expenditure; REE: Resting energy expenditure; TEF: Thermic effect of food, assumed to be 10% of total energy expenditure. (b) In the self‐reported approach, energy balance was calculated as the difference between daily energy intake from food records and total energy expenditure (in kcal/day). Calculated energy intake was computed as mean TEE between early and late rides + energy from changes in body energy stores (i.e., fat mass and fat‐free mass), divided by 23 days between assessments.

REE remained stable in the female athlete (17 kcal/day increase) and decreased by 146 kcal/day in the male athlete, (Figure 2a). Measured REE was close to predicted at both time points in the female cyclist (101.1%–102.5% of predicted) and remained higher than predicted in the male cyclist (118.1%–112.1% of predicted). TEE increased by 574 kcal/day in the female athlete, but decreased slightly by 97 kcal/day in the male. The increase in TEE for the female was primarily due to a 500 kcal/day rise in PAEE, while the male athlete experienced a relatively stable PAEE (58 kcal/day increase). PAL remained above 3.7 for both athletes during both periods, with the female's PAL ranging from 3.79 – 4.13, and the male's PAL ranging from 3.78 – 3.98.

### 3.4 Energy balance

Based on self‐reported energy intake, both cyclists exhibited a negative energy balance during each phase of the ride (Figure 2b), which became more pronounced in the female athlete (∆: −253 kcal/day) and less pronounced in the male athlete (∆: 898 kcal/day). Using calculated energy intake based on TEE and changes in body composition, the female cyclist maintained energy balance, whereas the male cyclist exhibited an estimated energy deficit of approximately 700 kcal/day.

## 4 DISCUSSION

This study is the first to assess energy balance in ultra‐endurance athletes consuming a plant‐based diet during a sustained, 30‐day effort. Our results show that both cyclists preserved or slightly increased fat‐free mass, and the female athlete maintained body weight despite substantial energetic demands. While additional health parameters were not assessed, these findings suggest that a plant‐based diet may support performance and minimize loss of fat‐free mass during ultra‐endurance cycling among recreational athletes.

Both cyclists maintained fat‐free mass, with body weight and composition changes comparable to or smaller than those reported in ultra‐endurance events lasting 12 h to 17 days (Knechtle, 2013). While evidence on muscle protein synthesis in athletes on plant‐based diets is mixed (Hevia‐Larraín et al., 2021), current recommendations suggest that consuming a variety of plant‐based proteins and sufficient energy can meet protein needs (Craig et al., 2009; Melina et al., 2016). For athletes with high energy demands, protein supplements may further support muscle protein synthesis (Shaw et al., 2022). Notably, both cyclists consulted a registered dietitian before the ride, which may have helped preserve fat‐free mass. The male cyclist gained fat‐free mass and lost fat mass—possibly due to sex‐based differences in metabolic adaptations to aerobic training. For example, in response to aerobic exercise, male rats tend to exhibit enhanced subcutaneous fat depletion due to increased aerobic metabolism and lipid utilization, whereas female rats demonstrate greater preservation of subcutaneous fat, likely influenced by greater adipogenesis and insulin signaling pathways (Many et al., 2024).

Food records suggested a negative energy balance in both athletes, which became slightly more pronounced in the female and appeared to improve in the male over time. Despite this, both maintained body weight throughout the ride, pointing to possible underreporting, intake variation between observations, or common self‐reporting errors such as underestimated intake due to database or labeling limitations. A well‐recognized limitation is the tendency to underestimate energy intake, with research suggesting athletes, on average, report about 20% less than actual intake, with greater inaccuracies occurring at higher intake levels (Capling et al., 2017). Given the athletes' extremely high TEE, it is unlikely that they consumed an excessive surplus between assessment periods. Given the challenges in accurately estimating dietary intake in field‐based settings (Stellingwerff et al., 2025), we calculated average energy intake using measured TEE and observed body composition changes. This approach suggested the female athlete consumed approximately 6000 kcal/day (closely aligning with her TEE), whereas the male demonstrated a deficit of ~700 kcal/day, suggesting an approximate average intake of 8300 kcal/day.

In multi‐week stage races, elite male cyclists consuming a presumed omnivorous diet typically report energy intakes of 5400–6500 kcal/day (Heikura et al., 2019; Muros et al., 2022; Rehrer et al., 2010). While the male athlete in this study self‐reported similar energy intake, calculations based on TEE and changes in energy stores indicated substantially higher intake than previous literature. This discrepancy may reflect limitations of self‐reported data or true greater intake due to longer daily cycling durations, larger body size, and a high proportion of fat‐free mass. Both athletes' energy intake appeared to scale with daily riding distance, suggesting intake was modulated—consciously or unconsciously—in response to exertion. This may reflect homeostatic regulation of energy balance or simply more eating time associated with longer rides. Although plant‐based diets are generally linked to lower energy intakes due to reduced dietary energy density (International Atomic Energy Agency, 2009), both athletes had followed plant‐based diets for several years and received dietary guidance prior to the ride. These factors may have supported behavioral and nutritional strategies enabling them to meet the extreme energy demands of ultra‐endurance cycling, underscoring the viability of high energy intake on a plant‐based diet.

Protein intake during the ride ranged from 13.2% to 17.3% of total energy intake (2.3–2.6 g/kg/body weight), slightly below some previous research in elite cyclists (Heikura et al., 2019; Muros et al., 2022), but similar to others (Areta et al., 2024). Both athletes regularly used a vegan protein supplement and consumed a variety of plant‐based protein sources (Data S2). This intake may have contributed to the preservation of fat‐free mass observed in the study. While precise protein requirements for such extreme events remain unclear due to the unprecedented nature of this context, these findings suggest that a well‐planned plant‐based diet can provide sufficient protein to support ultra‐endurance performance. Further research is needed to better understand protein metabolism, digestibility, and the impact of plant‐based diets on muscle protein balance in this context.

REE remained stable in the female athlete and slightly decreased in the male athlete pre‐ to post‐ride. This decrease is unlikely due to body composition changes, as adipose tissue contributes minimally to REE (Elia, 1992) and the male athlete also experienced a slight increase in fat‐free mass. Instead, the decline may reflect metabolic adaptations to prolonged ultra‐endurance exercise, such as reduced sympathetic activity, transient thyroid hormone changes, or shifts in glycogen and fluid balance (Burke et al., 2017; Ciloglu et al., 2005; Meyer et al., 2024). Improved skeletal muscle efficiency may also lower REE post‐exercise. The lack of significant REE changes suggests that inflammation or immune activation—often seen after shorter ultra‐endurance events (Comassi et al., 2015; Rubio‐Arias et al., 2019) or during periods of over‐reaching in training (Gleeson, 2007; Nieman, 2007)—either did not occur or had resolved within 2 days post‐ride, allowing a return to baseline metabolic function.

The female athlete experienced a substantial increase in TEE of >500 kcal/day from early to late ride, primarily due to increased PAEE. The reason for this increase is unclear, as PAEE would be expected to decline in the latter part of the ride due to slightly reduced daily mileage. However, it is possible that weather conditions (e.g., extreme wind or heat) may have contributed to elevated energy expenditure–particularly if they posed a greater relative physiological strain on the female athlete due to sex differences in body composition, thermoregulation, or absolute power output (Lim et al., 2011; Yanovich et al., 2020). It is also possible that differences in drafting durations between early and late stages of the ride contributed to changes in energy expenditure; however, this remains speculative, as drafting behavior was not systematically recorded. Menstrual cycle‐related hormonal fluctuations are unlikely to explain this change, as the athlete was likely in the early to mid‐follicular phase, when estrogen and progesterone levels are low. While energy expenditure is sometimes linked to higher progesterone levels (Benton et al., 2020), this was likely not a factor. The potential influence of a hormonal IUD, particularly shortly after insertion, remains unclear. Although increased non‐cycling activity could elevate PAEE, this seems improbable given minimal off‐bike movement. A more plausible explanation may be greater cycling effort during the latter part of the ride, though the underlying cause remains unknown.

Our results indicate that a PAL of approximately 4.0 (range: 3.78–4.13) is sustainable for non‐professional athletes over a 30‐day period. These values slightly exceed those reported in Race Across America runners over 5 days (mean PAL: 3.76 [range 3.08–4.13]) (Thurber et al., 2019), but are comparable to or slightly below those observed in professional cyclists during multi‐week races, such as the Giro d'Italia (4.37 ± 0.43 and 3.91 ± 0.39 using plateau and intercept techniques, respectively) (Plasqui et al., 2019) or Tour de France Femmes (4.32) (Areta et al., 2024). While the gastrointestinal system has been proposed as a limiting factor for sustained energy expenditure (Thurber et al., 2019), the athletes in this study were able to maintain energy balance and body composition, suggesting adequate digestive tolerance despite the high PAL. If the digestive tract indeed sets a limit for maximal energy intake (and consequently TEE) during prolonged activity, it is possible that a PAL of around 4.0 could be sustained for up to approximately 50–75 days (Thurber et al., 2019). However, a case study of a professional Ironman athlete sustaining a PAL of 3.4–3.8 over 3 years challenges previous assumptions (Dasa et al., 2024). Larger studies incorporating metabolic, gastrointestinal, and performance outcomes are needed to clarify the true limits of sustained human energy expenditure.

This study's strengths include the use of validated methods to assess energy balance and the novel focus on recreational athletes on plant‐based diets performing prolonged ultra‐endurance exercise. However, as with all case studies, generalizability is limited. Furthermore, DLW is more precise for groups rather than individual assessments (Melanson et al., 2018). Despite thorough instructions and verification procedures for dietary tracking, underreporting remains a possibility due to the inherent challenges of accurately capturing intake in real‐world settings (Capling et al., 2017; Ravelli & Schoeller, 2020). Another limitation is the absence of pre‐ride energy expenditure measurements, which limits our ability to compare energy expenditure and PAL during the ride to participants' habitual levels.

This study suggests that a plant‐based diet may be feasible to prevent substantial body composition changes despite high, sustained TEE during ultra‐endurance cycling. Further research involving larger and more diverse populations is needed to enhance our understanding of energy balance across various populations and contexts. Such studies could provide insights into optimizing performance and health in individuals with differing dietary preferences.

## AUTHOR CONTRIBUTIONS

Sarah A. Purcell, Edward L. Melanson, and Carla M. Prado conceived, designed, and conducted the research. Sarah A. Purcell, Edward L. Melanson, and Seth A. Creasy performed experiments. Sarah A. Purcell analyzed data, interpreted results of experiments, drafted the initial version of the manuscript, and edited and revised the manuscript. Sarah A. Purcell and Sarah A. Craven prepared figures. All authors approved the final version of the manuscript.

## FUNDING INFORMATION

Sarah A. Purcell and Carla M. Prado are partially supported by the Canada Research Chairs program (Government of Canada). Sarah A. Craven is supported by the Vanier Scholars Program (Government of Canada). This project did not receive any independent funding.

## CONFLICT OF INTEREST STATEMENT

Carla M. Prado has received honoraria and/or paid consultancy from Abbott Nutrition, Nutricia, Nestlé Health Science, Pfizer, Amra Medical, Novo Nordisk, and funding from Almased for unrelated research.

## ETHICS STATEMENT

The University of Alberta Research Ethics Board approved the study, and both participants provided informed written consent.

## Supporting information


Data S1.

Data S2.

## Data Availability

Data will be made available upon reasonable request to the corresponding author.
