# Supplementary material for: Energy balance in cyclists on plant‐based diets during a 30‐day, 4300‐km ride across Canada: Two case studies
Source: Physiol Rep. 2025 Nov 2;13(21):e70629. doi: 10.14814/phy2.70629 (PMC12580414; doi:10.14814/phy2.70629)
Supplement: Supplementary file 1 — Data S1. [file PHY2-13-e70629-s002.docx]

## ***Purcell et al. Energy balance in cyclists on plant-based diets during a 30-day, 4,300-kilometer ride across Canada: Two case studies***

## **Supplementary File 1: Pre- and post-ride assessments**

Participants were instructed to arrive at the Human Nutrition Research Unit in a fasted state, having abstained from caffeine for over 12 hours and from vigorous exercise for 24 hours prior to testing. Resting energy expenditure (REE) was measured using an open-circuit whole-room indirect calorimetry system, following the protocol previously described by our group (1, 2). Briefly, gas volume and concentration were recorded over a 60-minute period, with the initial 30 minutes serving as an acclimatization phase that was excluded from analysis. Oxygen (O₂) and carbon dioxide (CO₂) concentrations were measured every minute using differential analyzers (O₂: Oxymat 6, Siemens AG; CO₂: Advance Optima AO2000 Series, ABB Automation GmbH). Energy expenditure was calculated using the abbreviated Weir equation (3). The gas analyzers were calibrated weekly, including a “zero test” and span gas calibration, while the room calorimeter was calibrated prior to each assessment using premixed gas (20% O₂; 1% CO₂; balanced with nitrogen). Furthermore, a 24-hour propane burn test of the system is conducted quarterly to ensure accuracy, with pre-set ‘acceptable’ recovery ranges between 97-102%. REE was expressed in kcal/day and as a percent of predicted REE from the Mifflin St. Jeor equation (4).

Height was measured using a wall-mounted stadiometer. Total body water (TBW) was assessed using a tetrapolar, multi-frequency bioelectrical impedance analysis (BIA) (InBody 770; Seoul, South Korea). Participants removed all metal objects before testing. TBW obtained from the device was compared to TBW obtained from doubly labeled water (DLW) (described below).

Participants were provided with pre-weighed doses of stable isotopes for the DLW measurement. Participants also measured their body weight using a study-provided digital scale without shoes or heavy clothes on the morning of the DLW dosing. Each participant received a dose of 1.8 g/kg body weight of 10 atom% oxygen-18 (¹⁸O) and 0.12 g of 99.9 atom% deuterium (²H). A baseline urine sample was collected in the morning before the participants consumed the DLW dose. The dose was ingested through a straw, followed by rinsing the cup with tap water. Participants then provided a urine sample at 4.5 hours post-dose, as well as one sample per day at varying times for the following seven days (8 days total).

Isotope enrichments of ²H and ¹⁸O in the stock doses and urine samples were analyzed using off-axis integrated cavity output spectroscopy and isotope ratio mass spectrometry at the University of Colorado Anschutz Medical Campus (5). The natural logarithms of ²H and ¹⁸O enrichments were plotted against time with the slopes of the regression lines representing the rates of ²H and ¹⁸O elimination from body water (kH and kO, respectively). Dilution spaces for ²H (*N*_H_) and ¹⁸O (*N*_O_) were calculated by dividing the administered isotopes (in moles) by the intercepts of these regression lines. TBW was subsequently derived from the following equation (6, 7):

$$Total body water = 0.5 \times(N_{O}/c_{O} + N_{H}/c_{H})$$

Where c_H_ and c_O_ represent the ²H and ¹⁸O pool sizes relative to TBW.

To account for isotopic exchange with organic pools, nonaqueous c_H_ was set at 1.043 and c_O_ at 1.007. The isotope fractionation factor for ²H lost as water vapor was assumed to be 0.946 of the true water equilibration rate, while for ¹⁸O, the fractionation factor for carbon dioxide production was 1.038 of the true CO₂ production rate. Total energy expenditure (TEE) was determined using the two-point method outlined by Speakman *et al.* (8). Both the intercept and plateau method of calculating TEE are presented, although only the intercept method was used in our interpretation and calculation of components of energy expenditure and energy balance given the similar results from both approaches. A respiratory quotient of 0.88 was used for both measurement periods based on measured food quotient (see below). Physical activity energy expenditure (PAEE) was calculated as TEE (0.9) – REE, where the thermic effect of food was assumed to account for 10% of TEE. Physical activity level (PAL) was calculated as the ratio of TEE to REE (TEE/REE). Energy balance was assessed using two methods. First, it was calculated as the difference between mean daily energy intake from dietary records and TEE for each athlete and phase of the ride (‘self-reported’). Second, the intake-balance method was used, in which energy balance was estimated as the sum of each athlete’s mean TEE (early and late ride) to the change in body energy stores. Changes in body energy stores were determined from alterations in fat mass and fat-free mass from deuterium dilution, with energy coefficients of 9.3 kcal/g for fat mass and 1.1 kcal/g for fat-free mass (9). The total was then divided by 23 days to derive mean daily energy balance, representing the time between DLW measurements (‘calculated energy balance’).

Prior to their ride, participants received a food scale and instructions on recording dietary intake from a registered dietitian with extensive experience in nutrition research. In the weeks leading up to the ride, the cyclists had also worked with a separate registered dietitian to tailor their nutrition for the extreme demands of long-distance cycling, focusing on fueling, hydration, iron intake, and protein optimization for performance and muscle preservation on a plant-based diet. Participants maintained diet records for four continuous days at the beginning of each DLW collection period using paper forms; the cyclists were also encouraged to provide photos of their meals and all packaged goods before and after consumption. Dietary data were entered into FoodProcessor software (version 11.14.9; Trustwell, Salem, OR), with entries checked for accuracy by a member of the research team with extensive experience in dietary data entry. Energy intake was reported in kcal/day and kcal/kg/day. Macronutrient intake was expressed in absolute terms and as a percentage of total energy intake, with protein intake also reported in g/kg, as expressed in dietary guidelines. Food quotient was assumed to approximate respiratory quotient calculated as follows (10):

$$Food quotient = (protein \times0.81) + (fat \times0.71) + (carbohydrate \times1.00)$$

Where each macronutrient is the percent of energy contributed by each nutrient relative to the total energy intake (derived from applying the factors of 4, 9, and 4 for protein, fat, and carbohydrate, respectively).

**References**

1. **Purcell SA**, **Johnson-Stoklossa C**, **Braga Tibaes JR**, **Frankish A**, **Elliott SA**, **Padwal R**, **Prado CM**. Accuracy and reliability of a portable indirect calorimeter compared to whole-body indirect calorimetry for measuring resting energy expenditure. *Clin Nutr ESPEN* 39: 67–73, 2020. doi: 10.1016/j.clnesp.2020.07.017.

2. **da Silva BR**, **Pagano AP**, **Kirkham AA**, **Gonzalez MC**, **Haykowsky MJ**, **Joy AA**, **King K**, **Singer P**, **Cereda E**, **Paterson I**, **Pituskin E**, **Thompson R**, **Prado CM**. Evaluating predictive equations for energy requirements throughout breast cancer trajectory: A comparative study. *Clin Nutr* 43: 2073–2082, 2024. doi: 10.1016/j.clnu.2024.07.032.

3. **Weir JB**. New methods for calculating metabolic rate with special reference to protein metabolism. *J Physiol* 109: 1–9, 1949.

4. **Mifflin MD**, **St Jeor ST**, **Hill LA**, **Scott BJ**, **Daugherty SA**, **Koh YO**. A new predictive equation for resting energy expenditure in healthy individuals. *Am J Clin Nutr* 51: 241–247, 1990.

5. **Melanson EL**, **Swibas T**, **Kohrt WM**, **Catenacci VA**, **Creasy SA**, **Plasqui G**, **Wouters L**, **Speakman JR**, **Berman ESF**. Validation of the doubly labeled water method using off-axis integrated cavity output spectroscopy and isotope ratio mass spectrometry. *Am J Physiol Endocrinol Metab* 314: E124–E130, 2018. doi: 10.1152/ajpendo.00241.2017.

6. **Racette SB**, **Schoeller DA**, **Luke AH**, **Shay K**, **Hnilicka J**, **Kushner RF**. Relative dilution spaces of 2H- and 18O-labeled water in humans. *Am J Physiol* 267: E585-590, 1994. doi: 10.1152/ajpendo.1994.267.4.E585.

7. **Speakman JR**, **Nair KS**, **Goran MI**. Revised equations for calculating CO2 production from doubly labeled water in humans. *Am J Physiol* 264: E912-917, 1993. doi: 10.1152/ajpendo.1993.264.6.E912.

8. **Speakman JR**, **Yamada Y**, **Sagayama H**, **Berman ESF**, **Ainslie PN**, **Andersen LF**, **Anderson LJ**, **Arab L**, **Baddou I**, **Bedu-Addo K**, **Blaak EE**, **Blanc S**, **Bonomi AG**, **Bouten CVC**, **Bovet P**, **Buchowski MS**, **Butte NF**, **Camps SGJA**, **Close GL**, **Cooper JA**, **Creasy SA**, **Das SK**, **Cooper R**, **Dugas LR**, **Ebbeling CB**, **Ekelund U**, **Entringer S**, **Forrester T**, **Fudge BW**, **Goris AH**, **Gurven M**, **Hambly C**, **El Hamdouchi A**, **Hoos MB**, **Hu S**, **Joonas N**, **Joosen AM**, **Katzmarzyk P**, **Kempen KP**, **Kimura M**, **Kraus WE**, **Kushner RF**, **Lambert EV**, **Leonard WR**, **Lessan N**, **Ludwig DS**, **Martin CK**, **Medin AC**, **Meijer EP**, **Morehen JC**, **Morton JP**, **Neuhouser ML**, **Nicklas TA**, **Ojiambo RM**, **Pietiläinen KH**, **Pitsiladis YP**, **Plange-Rhule J**, **Plasqui G**, **Prentice RL**, **Rabinovich RA**, **Racette SB**, **Raichlen DA**, **Ravussin E**, **Reynolds RM**, **Roberts SB**, **Schuit AJ**, **Sjödin AM**, **Stice E**, **Urlacher SS**, **Valenti G**, **Van Etten LM**, **Van Mil EA**, **Wells JCK**, **Wilson G**, **Wood BM**, **Yanovski J**, **Yoshida T**, **Zhang X**, **Murphy-Alford AJ**, **Loechl CU**, **Melanson EL**, **Luke AH**, **Pontzer H**, **Rood J**, **Schoeller DA**, **Westerterp KR**, **Wong WW**. A standard calculation methodology for human doubly labeled water studies. *Cell Rep Med* 2: 100203, 2021. doi: 10.1016/j.xcrm.2021.100203.

9. **Racette SB**, **Das SK**, **Bhapkar M**, **Hadley EC**, **Roberts SB**, **Ravussin E**, **Pieper C**, **DeLany JP**, **Kraus WE**, **Rochon J**, **Redman LM**, **Group CS**. Approaches for quantifying energy intake and %calorie restriction during calorie restriction interventions in humans: the multicenter CALERIE study. *Am J Physiol Endocrinol Metab* 302: E441-8, 2012. doi: 10.1152/ajpendo.00290.2011.

10. **Black AE**, **Prentice AM**, **Coward WA**. Use of food quotients to predict respiratory quotients for the doubly-labelled water method of measuring energy expenditure. *Hum Nutr Clin Nutr* 40: 381–391, 1986.
